# DNA multi-bit non-volatile memory and bit-shifting operations using addressable electrode arrays and electric field-induced hybridization

**DOI:** 10.1038/s41467-017-02705-8

**Published:** 2018-01-18

**Authors:** Youngjun Song, Sejung Kim, Michael J. Heller, Xiaohua Huang

**Affiliations:** 1 Department of Bioengineering, University of California, San Diego, 9500 Gilman Drive, La Jolla, CA 92093 USA; 2 Department of Nanoengineering, University of California, San Diego, 9500 Gilman Drive, La Jolla, CA 92093 USA

## Abstract

DNA has been employed to either store digital information or to perform parallel molecular computing. Relatively unexplored is the ability to combine DNA-based memory and logical operations in a single platform. Here, we show a DNA tri-level cell non-volatile memory system capable of parallel random-access writing of memory and bit shifting operations. A microchip with an array of individually addressable electrodes was employed to enable random access of the memory cells using electric fields. Three segments on a DNA template molecule were used to encode three data bits. Rapid writing of data bits was enabled by electric field-induced hybridization of fluorescently labeled complementary probes and the data bits were read by fluorescence imaging. We demonstrated the rapid parallel writing and reading of 8 (2^3^) combinations of 3-bit memory data and bit shifting operations by electric field-induced strand displacement. Our system may find potential applications in DNA-based memory and computations.

## Abstract (web summary)

DNA based technology holds promise for non-volatile memory and computational tasks, yet the relatively slow hybridization kinetics remain a bottleneck. Here, Song et al. have developed an electric field-induced hybridization platform that can speed up multi-bit memory and logic operations.

## Introduction

DNA can be used to store digital information and to perform molecular computing due to the highly specific Watson-Crick pairings of nucleobases and the availability of molecular tools for parallel writing by chemical or enzymatic synthesis, copying by enzymatic replication, and reading by sequencing and hybridization-based detections such as fluorescence microscopy. The use of DNA-based computing to solve hard computation problems was first demonstrated by Adleman^1^ and further generalized by Lifton^2^ more than two decades ago. The huge parallelism inherent in DNA computing was realized by means of parallel hybridization and enzymatic reaction such as ligation as logical operations. Since then, various methods, including enzyme-free hybridization-based ones, such as toehold-mediated strand displacement technique have been developed to construct complex logic circuits to perform logical operations^3–8^, arithmetic calculations^4,9^, and even neural network computations^5^. Various mechanisms have also been proposed to implement DNA-based Turing machines^10,11^. However, the promise of using DNA computing to solve hard computational problems, intractable using conventional electronic computers, remains to be realized. Many challenges remain to be overcome^12,13^. These include slow operations due to hybridization kinetics and the inability for localized random access and manipulations of memory for efficient and fast computations^12.^

The tremendous potential of DNA as a medium to store memory or digital data, on the other hands, has been recently demonstrated^14–20^. The capacity, volumetric density, long-term stability, and energy efficiency of DNA memory are potentially far superior to conventional semiconductor-based memory devices^16,21^. However, access to DNA-based memory, usually in the form of DNA molecules in a solution, is a slow and laborious process, requiring DNA amplification and high-throughput sequencing. In contrast, modern electronic computers combine non-volatile memory and dynamic random-access memory (NVM and DRAM) and integrated circuits for localized arithmetic logic operations on memory to perform efficient and high-speed operations in reading and writing of data, and computations.

In this work, we investigated the relatively unexplored area of research on DNA-based memory and computing, where memory and logical operations are combined in a single platform for writing, reading, and computations. We used a microchip with a patterned array of individually addressable electrodes covered under a thin streptavidin-tethered hydrogel layer^22–28^ to enable rapid random-access activation and writing of data bits to memory cells, and bitwise manipulations of memory data. First, we demonstrated the basic writing operations by electric field-induced hybridization (EFH) and reading operation by single-channel fluorescence imaging of a single-level cell non-volatile memory (SLC-NVM) system. We then proceeded to demonstrate a tri-level cell non-volatile memory (TLC-NVM) system that is capable of parallel random-access writing of memory data by EFH and rapid optical readouts by three-channel fluorescence imaging. We showed the successful writing and reading of 8 (2^3^) combinations of 3-bit memory data. Finally, we investigated the ability to perform logical operations required for computations using the TLC-NVM system. We found that electric fields can be used to induce rapid strand displacement to perform bit shifting operations. Electric field-induced strand displacement (EFD) is much faster than the toehold-mediated strand displacement method commonly employed in other DNA-based logical circuits.

## Results

### DNA SLC-NVM

**Fig. 1:**
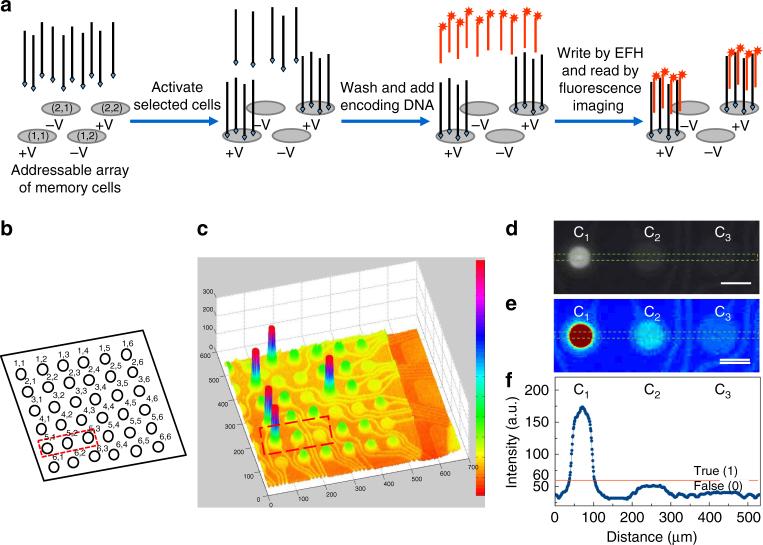
A DNA SLC-NVM system. **a** Random-access writing and reading operations using a microchip with an array of individually addressable electrodes. The specific cells are first selectively activated with the encoding ssDNA template molecules using the electrodes. The data bits of the cells are then written by EFH of fluorescently labeled bit-decoding DNA probes and read by fluorescence imaging. **b**,** c** A 6 × 6 SLC-NVM memory array. The addresses (**b**) and fluorescence image (**c**) of the array of cells after a writing operation had been performed on the selected cells. **d**–**f** Cell addressing specificity. Shown are the gray scale (**d**) and pseudo-color (**e**) fluorescence images, and fluorescence intensity (**f**) of three neighboring cells. Cell C_1_ was activated and written by EFH while cells C_2_ and C_3_ were not activated. A potential was applied to C_2_ but not C_3_ during the writing of data to C_1._ The bit status of cell C_1_ is true since its fluorescence intensity is above the threshold value. The bit status of the two neighboring cells C_2_ and C_3_ remains false since their fluorescence intensity is below the threshold value. Scale bars are 80 µm

Figure 1a shows the general operation of a DNA SLC-NVM system. A memory cell is first selectively activated by electrophoretic transport and affinity capture of encoding single-stranded DNA (ssDNA) template molecules to the specific cell location. This is carried out by injecting a solution containing the DNA into the microchip fluidic chamber, followed by the immediate application of a positive electric potential to the selected electrodes and a negative potential to the other electrodes using a patterned ring electrode array. The DNA molecules are captured on a streptavidin-hydrogel layer on the cell via streptavidin-biotin affinity binding. Unbound excess DNA molecules in the solution are removed by washing the fluidic chamber with a buffer solution. Each data bit is written by EFH of a bit-encoding fluorescently labeled oligonucleotide probe that is complementary to a segment of the immobilized encoding DNA template. The bit status of the cell is then read by fluorescence imaging.

We demonstrated the concept of random-access writing and reading operations of a 1-bit DNA NVM system using an array of 6 × 6 individually addressable electrodes (Fig. 1b, c). A 24-base biotinylated polydeoxyadenosine (biotin-dA_24_) was used as the encoding DNA template. Five cells with addresses of (1,2), (2,1), (3,4), (4,1), and (5,1) were activated by applying an electric potential (1.5 V) to the selected electrodes of the cells while a negative bias potential (−1.5 V) was applied to other addresses to simultaneously immobilize the encoding DNA template on the specific cells. The bit data of the cells were written by EFH of a fluorescently labeled 24-base polydeoxythymidine (Cy3-dT_24_) to the immobilized encoding DNA template molecules. The data were then read by fluorescence imaging. As can be observed in Fig. 1c, the fluorescence intensity at the addressed cells is much greater than that of other unaddressed cells, indicating that data bits at the specific cells were written and read successfully.

Next, we further investigated the addressing specificity of the random-access writing operations. Fig. 1d–f show the fluorescence image and intensity plots of three neighboring cells with addresses (5,1), (5,2), and (5,3) located inside the red box on the array shown in Fig. 1b. Cell (5,1) was selectively activated with biotin-dA_24_ using the random-access electrodes while the two neighboring cells (5,2) and (5,3) were not activated. The data bit of cell (5,1) was written by EFH of a Cy3-dT_24_ bit-encoding probe. During the writing process, an identical potential was also applied to cell (5,2), but not cell (5,3). The signal intensity at cell (5,3), about 41 (a.u., arbitrary unit), indicated the noise level due to background fluorescence. The relative signal intensity of about 51 at cell (5,2) is slightly higher than that of cell (5,3). This is likely due to non-specific binding of the Cy3-dT_24_ probe in addition to the general background signal. The signal intensity at cell (5,1) is about 171, which is much higher than those of cells (5,2) and (5,1). The specificity of addressing sub-arrays on the same microchip is shown in Supplementary Fig. 1. The results indicate that the memory cells can be activated by electric field-facilitated concentration and immobilization of the biotin-dA_24_ and written by EFH of Cy3-dT_24_ with a very high level of specificity without affecting the neighboring cells despite the presence of the DNA in the solution over the entire memory array. If a signal intensity value well above background signal, for example 60, is used as the threshold to define the true state (1) of the memory bit of the SLC, the data bit of the individual cells can be written with high accuracy. The use of an even higher threshold would minimize reading errors. The operation of our SLC-NVM is analogous to semiconductor SLC-NVM such as NOR flash memory. Data are stored in the activated cells while the unactivated cells serves as null memory, available for further use.

### DNA TLC-NVM

Three distinct fluorescence signals are used to encode the three data bits. Two approaches can be used to encode multiple bit signals. One approach is to immobilize different encoding DNA molecules on the same site. There are some disadvantages with this approach. These include reduced signal intensity due to the decreased number of encoding molecules per data bit, and the requirement for normalizing the amount of different encoding molecules. Another approach is to use different sequence segments on a single DNA molecule to encode the different bits^29^. This approach is advantageous over the first approach since it offers simplicity in operations and a higher signal per bit. In this work, we used this approach to demonstrate TLC-NVM and bit shifting operations using electric field-induced DNA strand displacement.

**Fig. 2:**
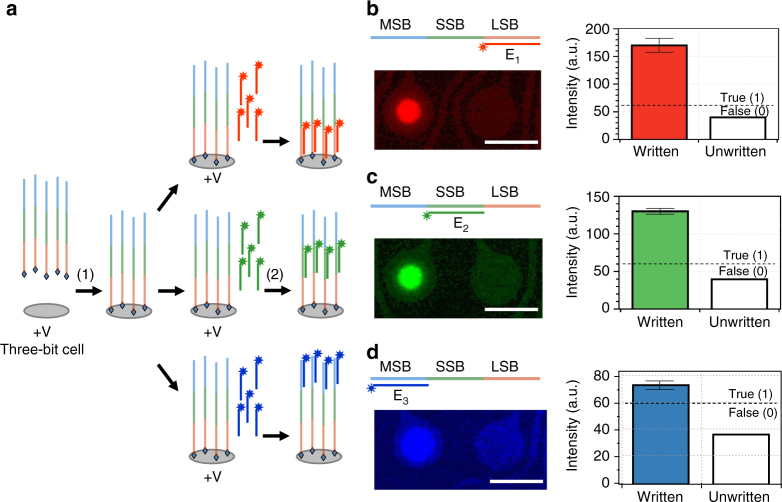
A DNA TLC-NVM system. **a** Writing operations of three-bit cells. The three bits are encoded by three different segments on a DNA sequence. Illustrated is the writing of three different bits, one on each cell. **b**–**d** Fluorescence readouts of the three bits, LSB (**b**), SSB (**c**), and MSB (**d**). Scale bars are 80 µm

First, we demonstrated the writing and reading operations of the individual bits of a 3-bit system. Three non-overlapping segments on a single DNA molecule (ES) were used to encode the three different bits: the least significant bit (LSB, 001), second significant bit (SSB, 010), and most significant bit (MSB, 100). Writing of data to the individual bits was performed by EFH of a bit-encoding oligonucleotide probe (E_1_, E_2_, or E_3_) labeled with a distinct fluorescence dye: Cy3-labeled E_1_ (red) for LSB, FAM-labeled E_2_ (green) for SSB, and Cy5-labeled E_3_ (blue) for MSB. The sequences of the encoding segments were designed to give a similar melting temperature (see Supplementary Table 1). For clarity, each bit was written separately on one cell. The basic operation is illustrated in Fig. 2a. Each cell was selectively activated by electric field-facilitated transport and immobilization of the biotinylated encoding DNA template molecules at the cell location. Each bit was written by EFH of a fluorescently labeled bit-decoding oligonucleotide probe whose sequence is complementary to the DNA segment encoding the bit. EFH was performed by applying a potential of 1.5 V to the electrode for 1.5 min. The data were read by fluorescence imaging. The results are shown in Fig. 2b–d. As can be observed, there is a variation in the signal intensity for the different bits and the cell size. The variations are due to the fluorescence properties of the different dye molecules and how their fluorescence signals are detected, and perhaps also some other factors that affect access and EFH to the different encoding segments of the DNA molecule. The variation in cell size is very likely due to the design and fabrication of the patterned electrode arrays (see Supplementary Fig. 2). However, the true state fluorescence signal intensity is always much greater than that of the background signal, allowing for the use of a high threshold to define the true state of the bits. As can be observed in Fig. 2c, d, the three individual bits were written and read successfully.

**Fig. 3:**
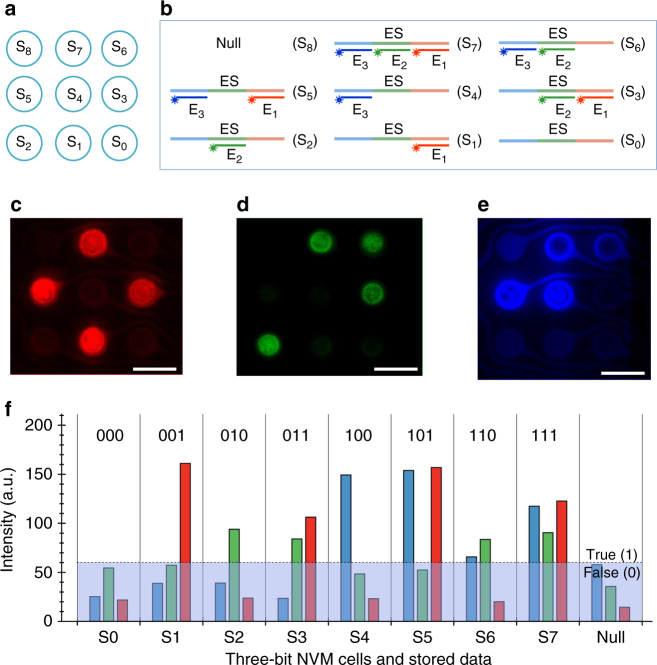
A DNA multi-cell TLC-NVM system. **a** The addresses of an array of eight individually addressable three-bit memory cells and a null cell (S_0_–S_8_). **b** Possible combinations of the three encoding bits. **c**–**e** Status of the E_1_ (**c**), E_2_ (**d**), and E_3_ (**e**) bits on the eight memory cells and the null cell. **f** Fluorescence intensity and written data of the eight memory cells. S_0_–S_8_: cell 1 to cell 9. ES: encoding sequence with three non-overlapping DNA segments, one for each of the three bits (LSB, SSB, and MSB). E_1_: Cy3-labeled DNA probe to encode LSB. E_2_: FAM-labeled probe to encode SSB. E_3_: Cy5-labeled probe to encode MSB. Scale bars are 160 µm

Next, we demonstrated the writing and reading of all 2^3^ combinations of three-bit memory data using an array of 3 × 3 electrodes on a microchip. The addresses of the array are designated as S_0_–S_8_ (Fig. 3a). All eight combinations of the three bits are illustrated in Fig. 3b. Each bit was written by EFH of the fluorescently labeled bit-encoding oligonucleotide, E_1_ for LSB, E_2_ for SSB, and E_3_ for MSS. The S_0_–S_7_ cells were written with three-bit data as 000, 001, 010, 011, 100, 101, 011, and 111, respectively. Cells S_0_–S_7_ were selectively activated while cell S_8_ was left unactivated as a null cell. The data were written in three steps. First, the LSB was written simultaneously onto S_1_, S_3_, S_5 _and S_7_ cells by EFH of E_1_. Second, the SSB was then written onto S_2_, S_3_, S_6_, and S_7_ cells by EFH of E_2_. Finally, the MSB was written onto S_4_, S_5_, S_6_, and S_7_ cells by EFH of E_3_. The data of the memory cells were then read by three-channel fluorescence imaging of the three different dyes (Cy3: red, FAM: green, and Cy5: blue) (Fig. 3c–e). There is a potential for bleed-through of the fluorescence signals due to spectral overlaps between the three dyes. With proper choice of excitation and optics, the bleed-through between the different channels can be minimized to below the threshold value (Supplementary Fig. 3).

Figure 3f shows the measured fluorescence intensities of the eight cells along with a null cell. It can be observed that the signal on the null cell (S_8_) remained low. This indicates the low non-specific binding due to random diffusion of the molecules from the solution in a single fluidic chamber covering the entire array during the three-step writing process. Even though the detected signal intensity and size of the cells are not uniform, the readouts are unambiguous if a proper threshold value is used to define the true (1) state of the bits. Therefore, we have clearly demonstrated that all eight combinations (2^3^) of a three-bit DNA NVM system can be written in parallel in a three-step EFH process and can also be read in parallel by three-channel fluorescence imaging.

### Bit shifting operations

**Fig. 4:**
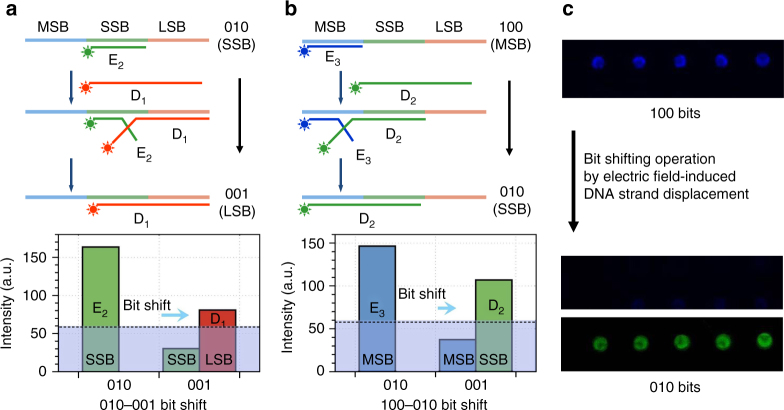
Bit shifting operations by electric field-induced DNA strand displacement. **a** Right shifting of 010 (SSB) to 001 (LSB) . **b** Right shifting of 100 (MSB) to 010 (SSB) . **c** Fluorescence readouts of bit shifting operations. The fluorescence  images show the readout data before (top panel, Cy5 channel) and after the parallel bit shifting (100–010) operation (middle panel, Cy5 channel, and bottom panel, FAM channel) on five three-bit cells

Bitwise manipulations are the fundamental operations of digital data storage and computations. In addition to multi-bit data storage using the DNA TLC-NVM system described above, here we demonstrated bit shifting operations on the same platform. The basic concept is illustrated in Fig. 4a, b. The operation is performed by the simultaneous erasing of one bit and writing of an adjacent bit by displacing the encoding DNA hybridized on the bit with another DNA encoding the adjacent bit using EFD. In one example (Fig. 4a), the SSB (010) was shifted to LSB (001) by replacing the bit-encoding DNA E_2_ (labeled with FAM) with a bit shifting DNA D_1_ whose sequence includes both the E_1_ and E_2_ encoding sequences, but labeled with the fluorescent dye (Cy3) used to encode E_1_. In another example (Fig. 4b), the MSB was erased by displacing the bit-encoding DNA E_3_ (labeled with Cy5) with a bit shifting DNA D_2_ whose sequence includes both the decoding E_2_ and E_3_ sequences, but is labeled with the fluorescence dye (FAM) used to encode E_2_. That resulted in the shifting of MSB (100) to SSB (010). Figure 4c shows the fluorescent images of five cells whose bits were shifted from 100 to 010. The writing of the bits was performed as described above for the operations of the DNA TLC-NVM. The electric field-induced hybridization and displacement was carried out similarly, by applying a 1.5 V potential for 1.5 min. Fluorescence readout of SSB to LSB bit shifting is shown in Supplementary Fig. 4.

## Discussion

We have shown a proof of concept of a DNA TLC-NVM system for data storage and bit shifting operations. We used a microchip with an array of individually addressable electrodes covered under a hydrogel layer to enable rapid and selective immobilization of encoding DNA template molecules onto the individual cells by electric field-facilitated transport, and fast parallel random-access writing operations by EFH of fluorescently labeled bit-encoding DNA molecules. The use of a hydrogel layer prevents the direct contact of DNA to the surface of the electrodes, preventing chemical damage to the DNA molecules. Parallel reading operation is enabled by multi-channel fluorescence imaging. Guided by computational modeling using the finite element method (detail in Supplementary Methods and Supplementary Fig. 5–6), we were able to optimize the conditions for efficient electrophoretic transport and electric field-induced hybridization using a histidine buffer system (Supplementary Fig. 8). We showed that rapid parallel operations, such as writing by EFH and bit shifting by EFD, can be performed on individual addressable cells with high specificity even though the entire array of memory cells are in contact with the DNA solutions in the fluidic chamber of the microchip. As a comparison, passive hybridization is slower (Supplementary Fig. 9). The hybridized bit-encoding DNA is robust and resistant to de-hybridization when a negative potential is applied to the electrode (Supplementary Fig. 10). Due to the digital nature of the data (0 or 1), potential readout errors could be minimized by using a threshold value well above the background signal to define the truth value of the data bits.

Using three segments on a single DNA molecule to encode three bits and three distinct fluorescence dyes (Cy3, FAM, and Cy5) to encode the bit signals, we demonstrated the parallel writing and reading operations of a TLC-NVM system. The bleed-through of fluorescence between the different dyes due to spectral overlaps was minimal (see Supplementary Fig. 3). We showed the successful parallel writing and reading of all eight (2^3^) combinations of three-bit data using an array of 3 × 3 memory cells. Bitwise manipulations (both arithmetic and logical) are the fundamental operations of digital computing. To explore the potential for DNA-based computing using logical operations, we demonstrated bit shifting operations in which the simultaneous erasure of a bit and the writing of an adjacent bit were performed by electric field-induced DNA hybridization and strand displacement.

We believe our DNA-based NVM system could be scaled up to higher number of bits per cell (e.g., five bits per cell) and for more complex logical operations in a relatively straightforward manner. A larger number of DNA segments on a single DNA template (e.g., five 20-base segments on a 100-base or longer DNA template molecule) could be used to encode more bits per cell. The maximum number of bits per cell that can be practically implemented is only limited by the ability to detect the different fluorescence dyes used to encode the different bits. The number of memory cells could also be significantly increased by using a CMOS-based microchip with much higher density of individually addressable electrode arrays^30^. More complex bitwise operations are also possible by using more sequences for bit shifting operations by EFD. In addition to editing the stored memory data, bit shifting operations can be used to perform logic gate functions and arithmetic operations. For example, the 010 (SSB) to 001 (LSB) bit shifting operation is equivalent to a subtraction operation.

The ability to store and manipulate memory data on a single platform on localized cells is a unique feature of our system. This offers many capabilities to be further explored for potential applications. With a higher number of bits per cell and more memory cells, the amount of data and logical operations scales exponentially. The writing, reading, and bit shifting (and perhaps other logic) operations will be essentially identical to what is described in this work. However, the number of operations scales only linearly, in terms of the number of steps to be performed and the number of fluorescence channels required for reading. Moreover, our microchip-based system is compatible with existing in situ short-read DNA sequencing chemistry such as the Illumina reversible terminator sequencing chemistry^31^. The implementation of such a readout method will enable very high degree of multiplexing capability for detection of the sequences used for encoding the multiple bits and additional bitwise operations for implementing AND, OR, NOT, and other logic gates. We believe that rewriting of memory data could be possible and rather straightforward.

As a technology for massive data storage, our system compares less favorably, in terms of cost and capacity, to other solution-based technologies that can make use of individual nucleobases for encoding and massive parallel sequencing for data decoding. However, surface-based systems offer certain advantages over solution-based systems. These include the ability to manipulate molecules in localized areas and direct on-chip readouts as we have demonstrated here. DNA computing based on passive DNA hybridization to random or ordered arrays with fixed addresses on the surface is not new and has been demonstrated by other researchers^3,32–34^. Our work further advances the surface-based method by using EFH and EFD to speed up the operations and using addressable electrode arrays to enable random-access parallel writing and bitwise operations. Our microfluidic-based system with addressable arrays could potentially be used to enable compartmentalization of DNA or nucleic acid memory by reducing the access volume and energy of operation^16^.

DNA-based complex logical circuits have been constructed to perform neural network computations, arithmetic computations, and to control chemical reaction networks^3–11^. Their operations commonly rely on passive hybridization and toehold-mediated strand displacement due to random molecular diffusion in solutions, which could take up to 30 min or longer per operational event due to the relatively low concentrations of reactive DNA components in solution. Complex computations could take hours to days^12^. Recently, it has been shown that the hybridization and strand displacement operations could be sped up significantly by using spatially co-localizing circuit elements on DNA origami, reducing computation time to minutes instead of hours required for circuits with diffusible components^35^. In this work, we have also shown that DNA hybridization and strand displacement can be dramatically accelerated by using EFH and EFD as compared to passive reactions. This is possible because the DNA molecules in solution can be electrophoretically transported, and thus concentrated, to the desired locations, and alteration of the ionization state of the histidine molecules in our unique buffer near the electrodes significantly enhances hybridization kinetics^23,28,36^. In our TLC-NVM system, both parallel writing and bit shifting operations can be performed in less than 1.5 min. Read operation by fluorescence imaging is also parallel and rapid, taking only a few seconds.

In this work, we used prefabricated microchips available to us (NanoChip^®^ 100 by Nanogen Inc.) and a conventional epifluorescence microscope for imaging. Relative high concentrations of DNA were used for ease of imaging to demonstrate the basic concepts. It has been well documented that DNA with nanomolar concentrations can be concentrated and hybridized in seconds using electrophoretic transport and electric field-induced hybridization^30^. It has also been demonstrated that EFH enabled nanomolar target DNA solution to be detected with high signal to noise (3:1) using higher density CMOS-based microchips (up to 10,000 sites) with integrated waveguide laser illumination and CCD camera^30^. We believe it is possible to reduce the operation cost of our DNA MLC-NVM systems by using higher-density arrays with individually addressable random-access memory cells and lower concentrations of DNA components. Interestingly, high-density arrays of oligonucleotides can also be synthesized in situ using microchips with arrays of individually addressable electrodes covered under a permeation layer^37–40^. Such microchips could potentially be utilized to enable scaling up and cost-effective implementation of our DNA multi-bit NVM systems. Our future work will explore this possibility.

In conclusion, we have developed a DNA TLC-NVM system that is capable of parallel random-access writing of memory and bitwise operations by EFH and EFD, and rapid readouts in one single platform. Our surface-based microfluidic system also enables compartmentalization for faster addressing and other localized operations. These unique capabilities could be further improved and used to enhance or advance other existing methods for DNA-based data storage or memory which in recent years have been demonstrated to be far more superior to semiconductor-based technologies in term of data density and energy efficiency. The demonstrated EFD method could also potentially be utilized to accelerate the operations of DNA-based complex logical circuits that rely on DNA hybridization and strand displacement.

## Methods

### Materials

All oligonucleotides were purchased from Integrated DNA Technologies, Inc. The sequences are listed in Supplementary Table 1. 1× TBE (Tris-Borate-EDTA) buffer was prepared as a solution of 0.089 M Tris, 0.089 M boric acid, and 0.002 M EDTA, pH 8.3. The conductivities of buffer and salt solutions were measured using a conductivity meter (Accumet Research AR-50, Fisher Scientific). The conductivity of the hydrogel layer was estimated according to the literature^36,40^. Microchips with an array of 100 addressable electrodes from Nanogen were used as a platform for the prototype DNA NVM systems. To control the individual electrodes, the microchip was interfaced via a pogo-pin connector to an array of single pole double throw (SPDT) switches on a printed circuit board (PCB). Fluorescence imaging was performed using either 5×/NA0.12 or 10×/NA0.3 objective and an epifluorescence microscope (DMRBE, Leica Microsystems Inc.) equipped with a CCD camera (ORCA-ER, Hamamstsu Inc.). A 100 W Hg arc lamp was used as excitation source. The Cy3, FAM, and Cy5 channels were imaged using three different filter cubes (center wavelength/bandwidth of bandpass filters for excitation (ex) and emission (em): Cy3, 545 nm/20 nm (ex) and 605 nm/70 nm (em); FAM, 480 nm/30 nm (ex), 535 nm/50 nm (em); Cy5, 620 nm/50 nm (ex), 700 nm/75 nm (em)).

### DNA SLC-NVM writing and reading operations

To activate the specific addresses or memory cells, the encoding ssDNA template molecules were selectively immobilized on the streptavidin covalently attached to a 2% hydrogel matrix above the locations of the cells. This was performed by injecting a solution of 20 µM biotin-dA_24_ in a buffer containing 10 mM NaCl and 50 mM histidine (His-Buffer) into the fluidic chamber of a Nanogen 100-site microchip, and then immediately applying a 1.5 V potential to the individually addressable electrodes using the SPDT switches on the PCB for 1.5 min. The fluidic chamber was then washed using 1× TBE buffer. To write the bits on the selected cells by EFH, a 1.5 V potential was applied to the selected individual electrodes for 1.5 min after a 20 µM of decoding Cy3-dT_24_ in His-Buffer was injected into the fluidic chamber. The chamber was then washed immediately using 1× TBE. To read the bit information of the cells by fluorescence microscopy, the surface of the microchip was imaged using a Cy3 filter cube.

### DNA TLC-NVM writing and reading operations

A DNA molecule that contains three segments with different sequences is used to encode three bits. The sequences of the encoding template DNA molecule (ES) and the bit-encoding fluorescently labeled DNA molecules (Cy3-labeled E_1_, FAM-labeled E_2_, and Cy5-labeled E_3_) are listed in Supplementary Table 1. The specific cells were activated by immobilizing the encoding biotinylated ssDNA molecules (biotin-ES) on the hydrogel at the cell locations using the same procedure as described for the SLC-VNM system. To demonstrate the writing and reading operations of the three individual bits, one at a time on three separate cells, only a small portion of a 100-site Nanogen microchip was used. The three bits were written sequentially as follows. The 001 bit (LSB) was written on one cell by injecting 20 µM E_1_ in His-Buffer and then applying 1.5 V to the cell electrode for 1.5 min. The fluidic chamber was then washed immediately using 1× TBE. The 010 bit (SSB) and 100 bit (MSB) were written to two separate cells using E_2_ and E_3_, respectively, in the same manner. The data were read by three-channel fluorescence imaging using the filter sets for Cy3, FAM, and Cy5.

A 3 × 3 array of cells with individually addressable electrodes on a 100-site Nanogen microchip was used to demonstrate the random-access writing and reading of 2^3^ combinations of data bits in a DNA TLC-NVM system. To write the LSB on addresses S_1_, S_3_, S_5_, and S_7_ (Fig. 3a) in parallel, EFH was performed by injecting 20 µM E_1_ in His-Buffer and then applying 1.5 V to the selected electrodes for 1.5 min. After the chamber was washed using 1× TBE, the SSB on addresses S_2_, S_3_, S_6_, and S_7_ (Fig. 3a) was written similarly in parallel using 20 µM E_2_. The MSB was written on addresses S_4_, S_5_, S_6_, and S_7_ using 20 µM E_3_ in the same manner. After the chamber was washed, the data on the cells were read by three-channel fluorescence imaging.

### Bit shifting operations by electric field-induced hybridization and displacement (EFD)

The same TLC-NVM system was used. The additional fluorescently labeled DNA sequences (Cy3-labeled D_1_ and FAM-labeled D_2_) for the bit shifting operations are listed in Supplementary Table 1. Immobilization of the encoding ssDNA (ES) on the addresses and writing operations were performed in the same manner as described above. First, we demonstrated MSB (100) to SSB (010) bit shifting. MSB (100) bit was written on the selected addresses using E_3_, followed by a wash with 1× TBE. A solution of 20 µM switching DNA D_2_ in His-Buffer was injected into the chamber, and then a 1.5 V potential was applied to the same set of electrodes for 1.5 min to effect the bit shifting by EFD. The effect was read by fluorescence imaging using both FAM and Cy3 channels. Second, we also demonstrated SSB (010) to LSB (001) bit shifting. The SSB bit was written using E_2_ and shifted using D_1_ in a similar manner.

### Image processing and data analysis

The gray scale images were processed and analyzed using ImageJ (NIH) and MATLAB (Mathworks). The plots were prepared using Origin 2015 (OriginLab Corp) and Datagraph 4.0 (Visual Data Tools, Inc.).

### Computational simulations

To gain some insights into the EFH process and to optimize the operations of our DNA NVM system, we used the finite element method to simulate the electric field strength and distribution, and the electric field-induced distributions of histidine, Na^+^, Cl^−^, and DNA on a Nanogen NanoChip^®^ with an array of 100 individually addressable electrodes. Detail of the simulations is provided in the Supplementary Methods.

### Data availability

The data sets generated during and/or analyzed during the current study are available from the corresponding author on reasonable request.

## Electronic supplementary material

Supplementary Information
