# Supplementary material for: DNA multi-bit non-volatile memory and bit-shifting operations using addressable electrode arrays and electric field-induced hybridization
Source: Nat Commun. 2018 Jan 18;9:281. doi: 10.1038/s41467-017-02705-8 (PMC5773625; doi:10.1038/s41467-017-02705-8)
Supplement: Supplementary file 1 — Supplementary Information [file 41467_2017_2705_MOESM1_ESM.pdf]

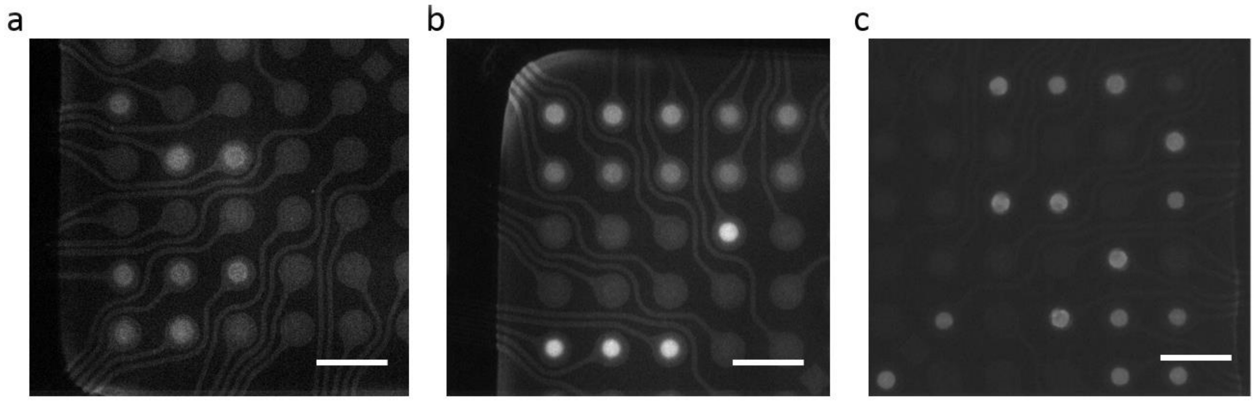

Supplementary Figure 1. Random-access addressing specificity of SLC-NVM system. Fluorescence images of three sub-arrays of memory cells on the same 100-site microchip. The bright circular spots are the cells to which a data bit has been written by EFH of Cy3-labeled dT_{24}. **a**, 6x6 memory cells on the bottom left portion of the chip. **b**, 5x5 memory cells on the top left portion of the chip. **c**, 6x6 memory cells on the top right portion of the chip. Scale bars are 160 µm. The average signal intensity of the addressed cells is about 111 (a. u.). The average background signal intensity of the un-addressed cells is about 47 (a. u).

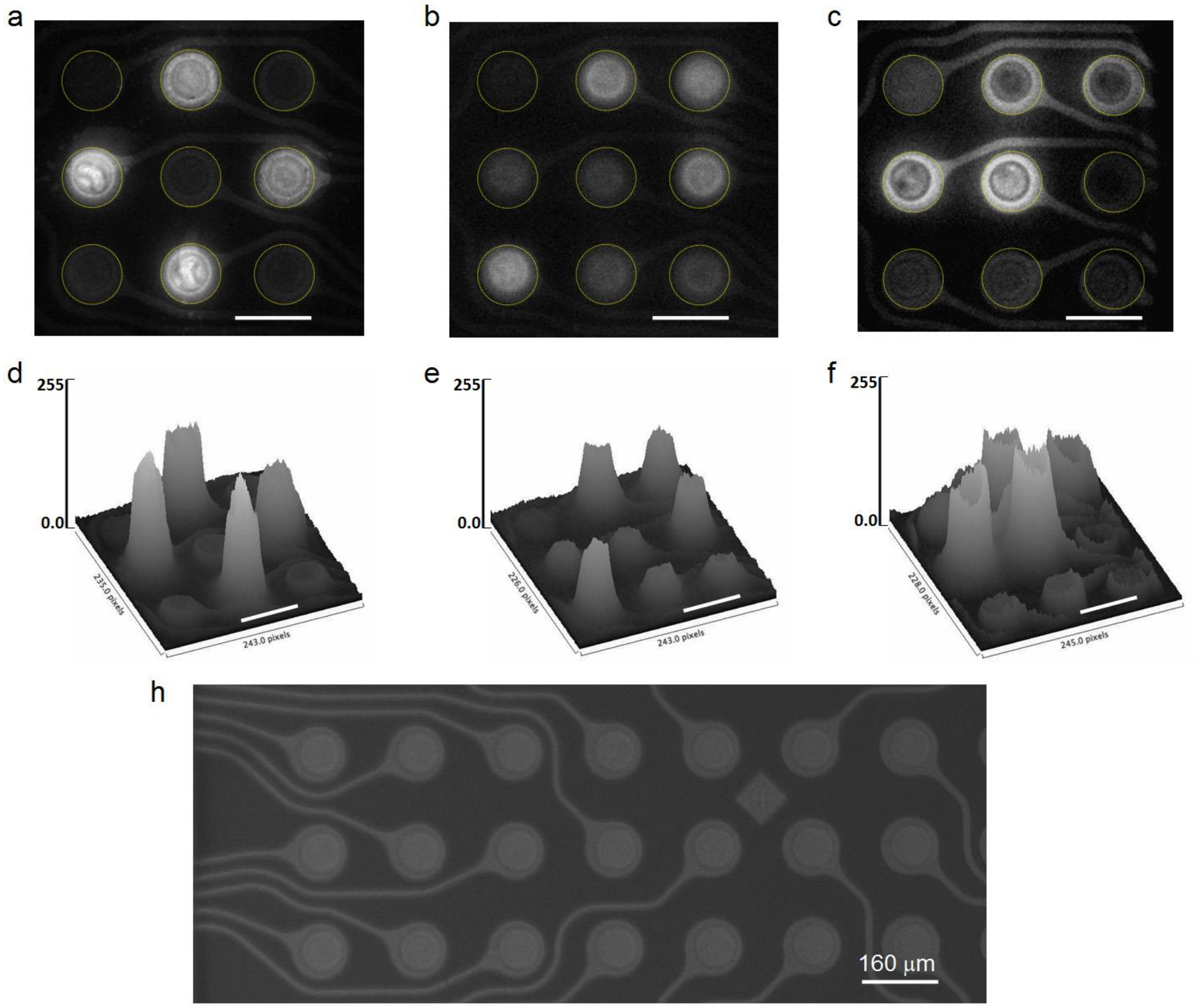

Supplementary Figure 2. Variations in signal intensity and cell size in a TLC-NVM system. The grey-scale fluorescence images and 3D intensity plots of Fig. 3c-e. in the main text. **a-c**, Fluorescence images of Cy3 (**a**), FAM (**b**) and Cy5 (**c**) channels, respectively. The intensity value for each cell was obtained by averaging the signal intensity of the pixels outlined by the yellow circle. **d-f**, 3D plots of the signal intensity in (**a**), (**b**) and (**c**), respectively. **h**, Bright-field image of a portion of the electrode array on the microchip. Scale bars are 160 µm.

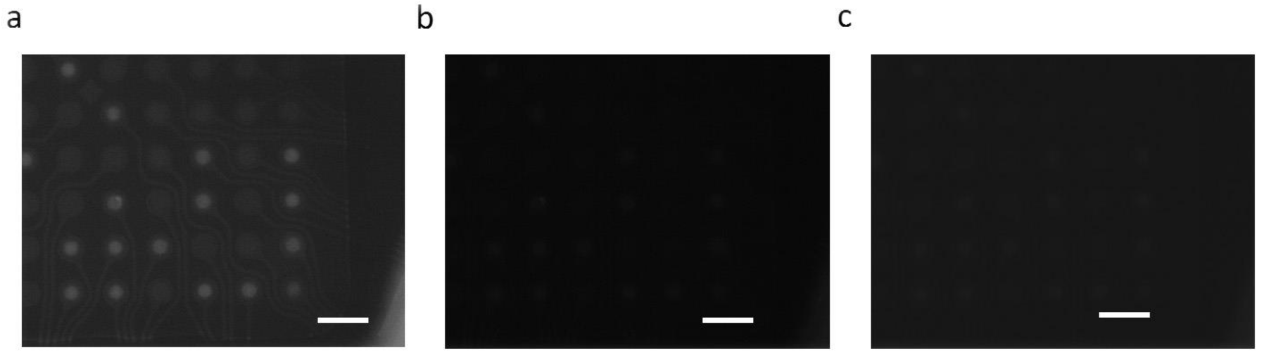

Supplementary Figure 3. Characterization of signal bleed-through due to spectral overlaps. A TLC-NVM array in which data were written only to the SSB by EFH of FAM-labeled E_2 and read by three-channel fluorescence imaging. The fluorescence images are shown. **a**, FAM channel. **b**, Cy3 channel. **c**, Cy5 channel. The average signal intensity of the written cells in the FAM, Cy3 and Cy5 channel is 65.6 (a. u.), 14.1 (a. u.) and 25.2 (a. u.), respectively. Scale bars are 160 µm.

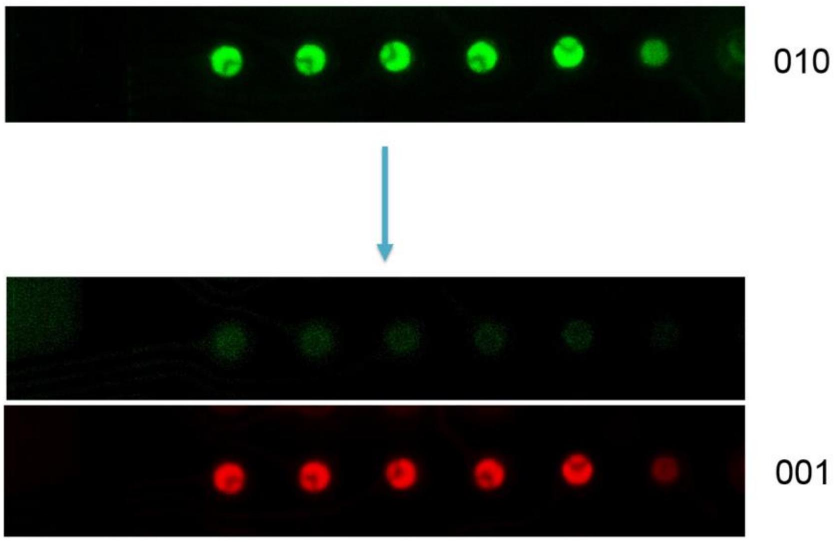

Supplementary Figure 4. SSB to LSB bit shifting operation. A fluorescent images showing bit information on 5 addresses. Top panel: memory cells written with SSB as indicated by a signal in the green (FAM) channel. Middle and bottom panels: SSB to LSB bit shifting as indicated by the disappearance of the green (FAM) signal (middle panel) and the concomitant appearance of red (Cy3) signal (bottom panel).

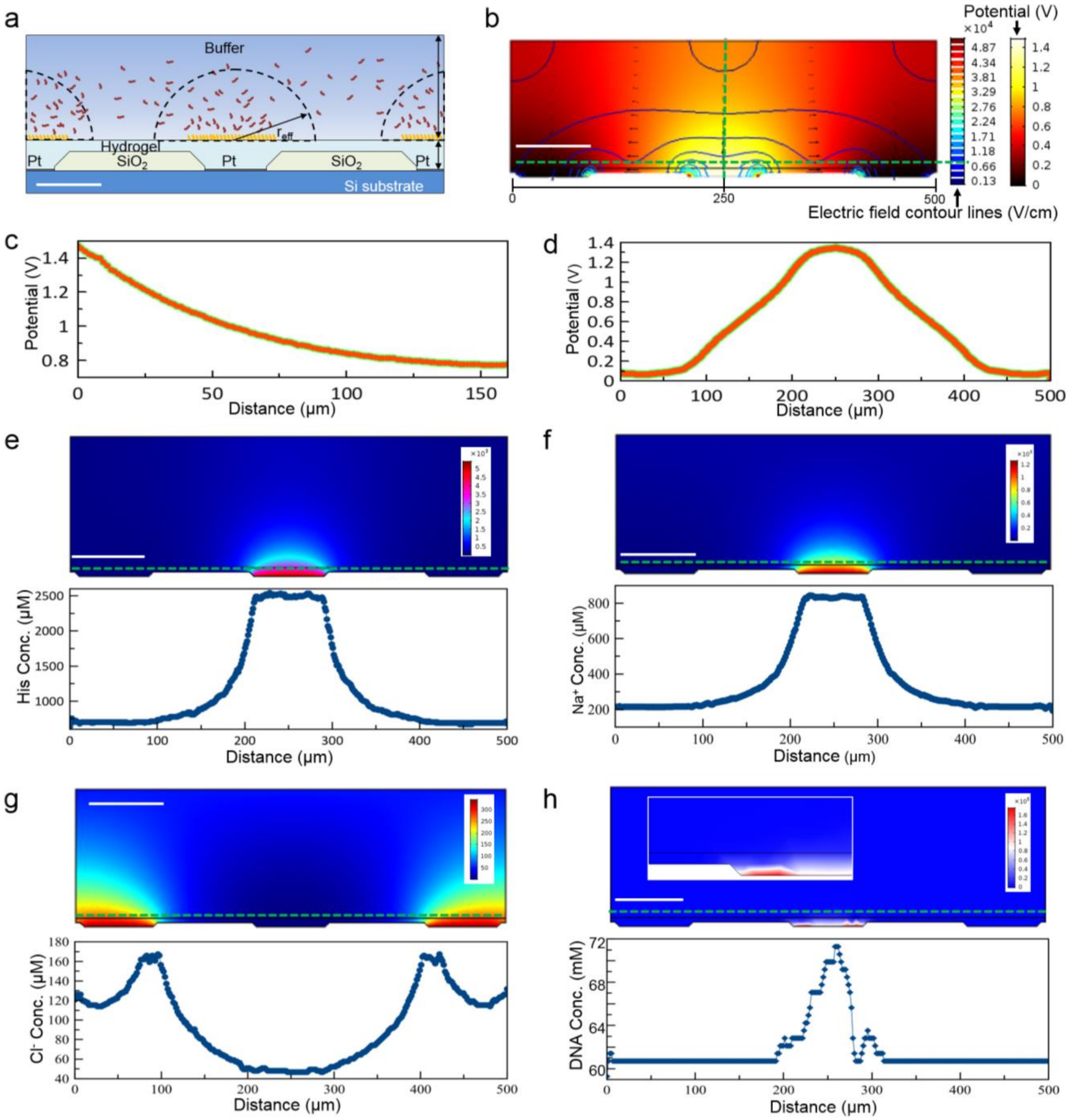

Supplementary Figure 5. Computational simulations of electric field-induced distribution of ions and molecules. a, A cross section of a portion of the chip used for electric field-induced hybridization. r_{eff} : effective radius of cell. Yellow/red strings: immobilized DNA molecules in a 10 μm 2% agarose hydrogel layer. **b**, Electrical field and potential distribution. The electrical field distribution is indicated by the color contour lines while the potential distribution is indicated by the color map. Left color bar: color map for electric field strength contour lines (in V/cm). Right color bar: electric potential color map (in V). **c**,**d**, intensity along the vertical (**c**) and horizontal (**d**) green dashed lines in **b. e-h**, Spatial distribution of histidine molecules (**e**), Na^+ (**f**) and Cl^- (**g**) ions, and DNA (**h**) under applied electric fields. The plots show the intensity profiles along the horizontal green dashed lines in the intensity heat maps. Scale bars are 80 μm.

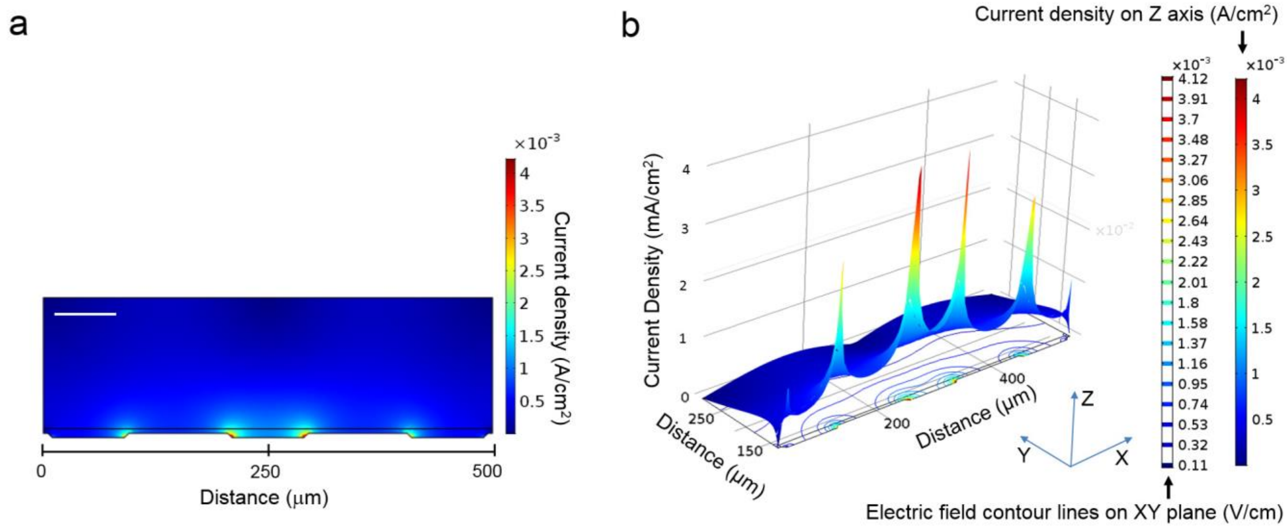

Supplementary Figure 6. Current density profiles from FEM simulations. **a**, 2D plot of current density (A/cm^2) when a 1.5 VDC is applied to the central electrode while the other two adjacent electrodes were maintained at 0 V. **b**, 3D plot of current density. The current density is plotted along the vertical axis. The distance is plotted along the X-Y plane. Left color bar: color map for the electric field strength contour lines on the XY plane (in V/cm). Right color bar: color map for the current density along the Z axis (in A/cm^2). Scale bar is 80 μm.

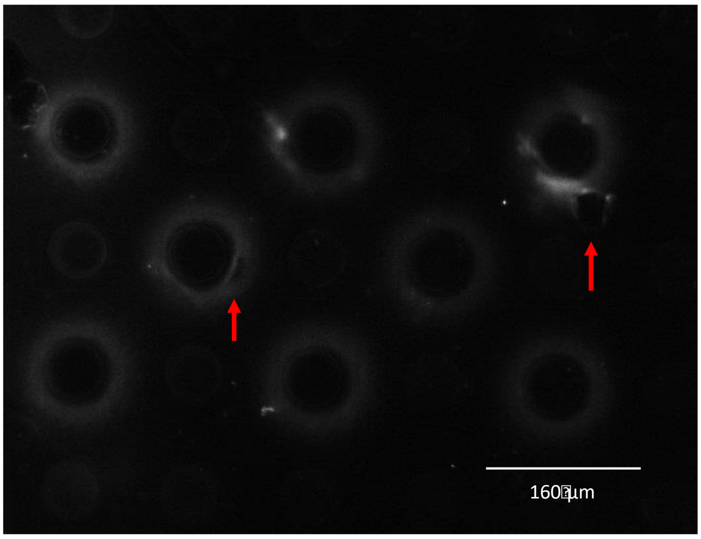

Supplementary Figure 7. Detachment of hydrogel layer due to electrochemical damage when a high potential is applied to the electrodes. The application of a high applied potential (2.5 VDC) resulted in excessive hydrolysis of water and electrochemical damage to the hydrogel layer. Fluorescence image of a portion of the microchip after a high voltage (2.5 VDC) was applied to the selected electrodes for 1.5 minutes. The detachment is indicated by the red arrows.

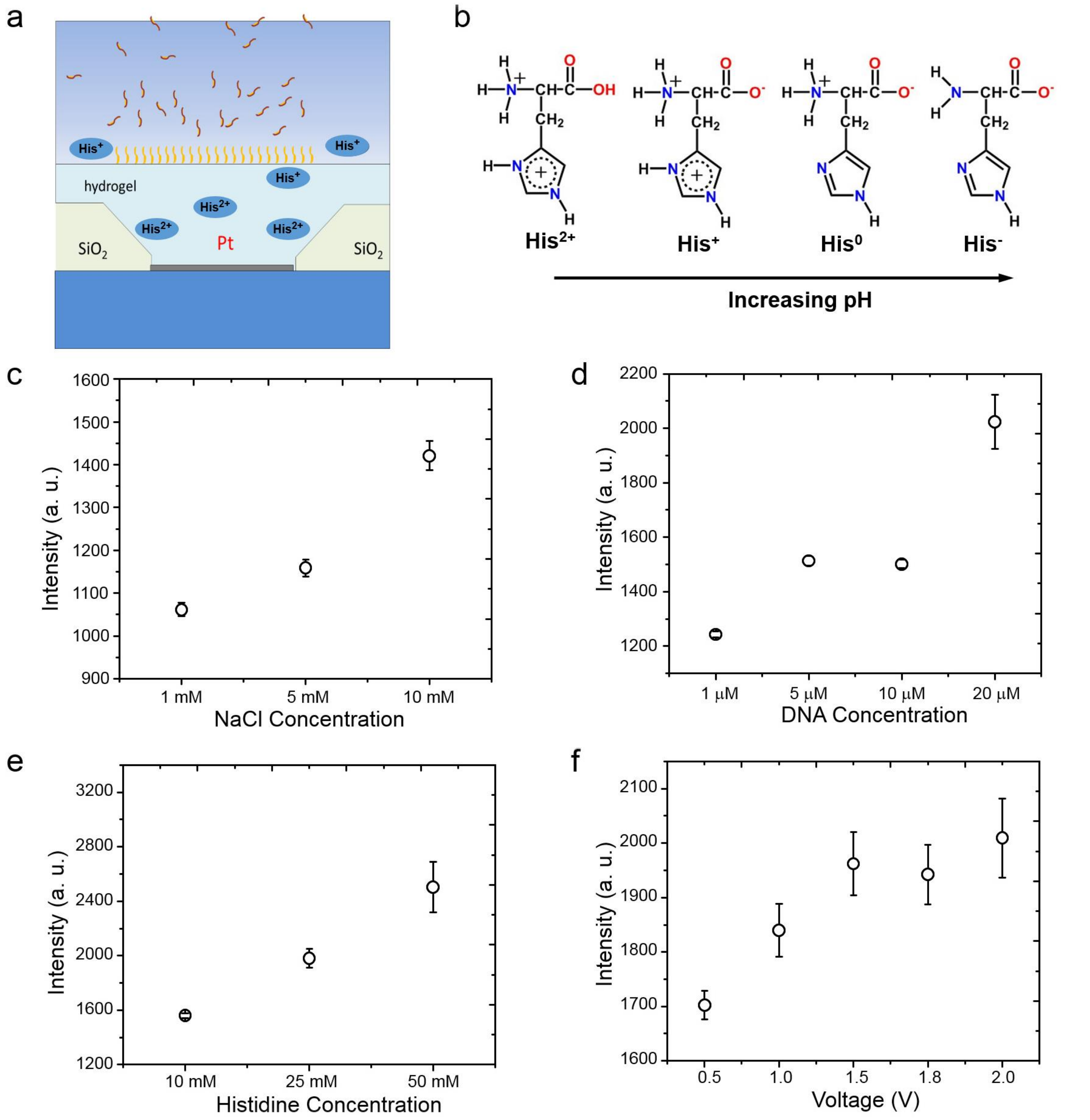

Supplementary Figure 8. Experimental optimization of electrical field-induced hybridization. a, A schematic illustrating the electrode, hydrogel layer, histidine ions, and DNA molecules on one cell. **b,** Ionization states of histidine molecules as a function of pH. **c-f,** Fluorescence intensity of one cell where electric field-induced DNA hybridization was performed under various concentrations of NaCl (**c**), DNA (**d**), histidine (**e**), and applied voltages (**f**). Four measurements were performed for each concentration or voltage. The average values are represented by the circles. The standard deviations of the measurements are indicated by the error bars.

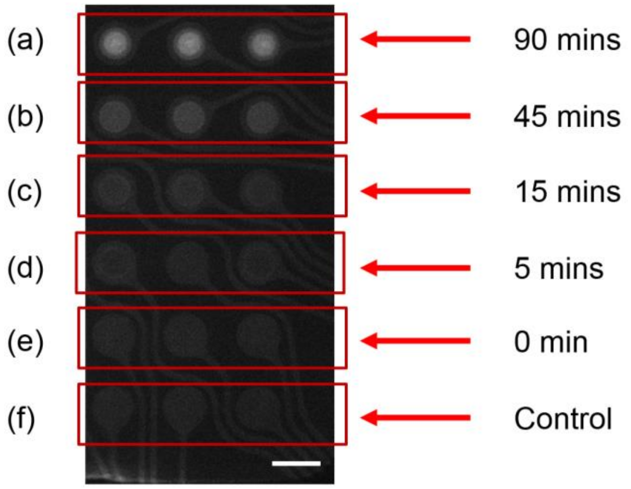

Supplementary Figure 9. Writing by passive hybridization. The cells were activated with biotin-dA_{24} by applying 1.5 VDC for 1.5 minutes at the selected rows. The hybridization of Cy3-dT_{24} was performed passively. **Rows a-e**: passive hybridization for 90, 45, 15, 5 and 0 minutes, respectively. **Row f**: control cells not activated with template DNA. Scale bar is 80 µm. The same microchip was used for the time-serial passive hybridization experiment. First, row a was activated with 20 µM biotin-dA_{24} and washed with His-Buffer. Then a solution of 20 µM Cy3-dT_{24} was injected into the entire fluidic chamber. The hybridization was allowed to proceed for 45 minutes at room temperature and the solution was then flushed away with His-Buffer. Second, in a similar manner, row b to row e was activated sequentially and hybridization with 20 µM Cy3-dT_{24} solution in the entire chamber was allowed to proceed for 30, 15, 5 and 0 minutes, respectively. Finally, after washing with His-Buffer, fluorescence imaging in the Cy3 channel was performed. The average relative fluorescence intensity of the cells after passive hybridization for 0, 5, 15, 30, 45 and 90 minutes is 33.3, 36.2, 35.6, 42.6, 58.2 and 103.9, respectively. Scale bars are 160 µm.

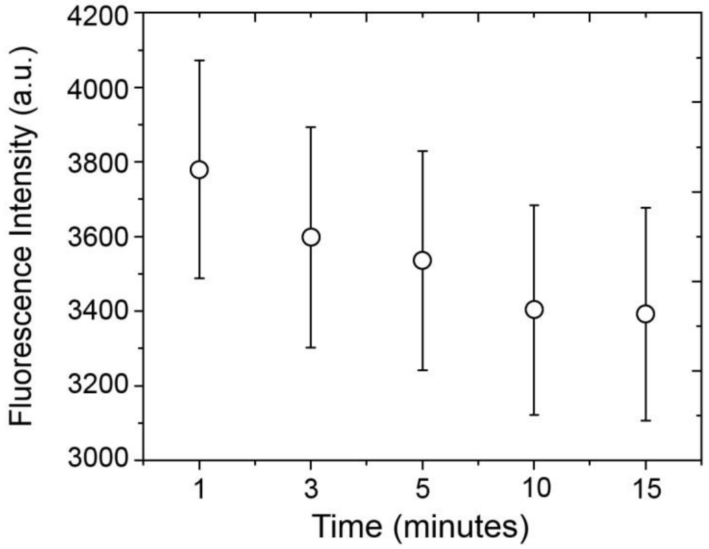

Supplementary Figure 10. Stability against de-hybridization by negative electric potentials.

A cell in a SLC-NVM system was activated with biotinylated dA_{24} and written by EFH of Cy3-dT_{24} in His-Buffer. A -1.5 VDC was applied to the cell and fluorescence intensity was monitored over time to quantify de-hybridization. The intensity values from four cells were used to calculate the average values and standard deviations. The average values are represented by the circles. The standard deviations are indicated by the error bars.

Supplementary Table 1. DNA sequences and melting temperatures (Tm)

Encoding DNA	Sequences	Tm (°C)* (1 μM)	Tm (°C)* (20 μM)
Biotin-dT_{24}	Biotin-TTTTTTTTTTTTTTTTTTTTTTTT-3’	43.4	46.7
Cy3-dA_{24}	Cy3-AAAAAAAAAAAAAAAAAAAAAAAA-3’	43.4	46.7
ES	Biotin- TTATTCATTTTGGTTTTCATTGTTACGCAGGACAGACAGG-3’	62.6	65.7
E_1	Cy3-AAAACCAAAATGAATAA-3’	40.0	44.8
E_2	FAM-TGCGTAACAATG-3’	41.7	45.1
E_3	Cy5-CCTGTCTGTCC-3’	40.4	47.7
D_1	Cy3- TGCGTAACAATGAAAACCAAAATGAATAA-3’	56.3	59.2
D_2	FAM-CCTGTCTGTCCTGCGTAACAATG-3’	59.6	63.3

*Tm was calculated at 50 mM monvalent salt (Na^+) using OligoAnalyzer 3.1 (at URL http://www.idtdna.com/calc/analyzer/, Integrated DNA Technologies Inc.).

Supplementary Methods

Computational simulations of electric field-facilitated transport of ions and molecules

To gain a better understanding of the EFH process and to optimize the operations of our DNA NVM systems, we simulated the electric field strength and distribution, and the electric field-induced distributions of histidine molecules, Na^+, Cl^- and DNA molecules over the cells of a Nanogen microchip with an array of 100 individually addressable electrodes. The simulations were performed using the COMSOL Multiphysics 5.2 software package (COMSOL Inc.).

Supplementary Fig. 5a shows a cross-sectional structure of one cell and its vicinity on the microchip. The thickness of the platinum electrodes is 200 nm. The diameter of the exposed area of the ring electrode is 80 μm. Except the exposed area of the ring electrodes, the surface is covered with a 20 μm thick SiO_2 insulator layer. The entire surface is covered underneath a 10 μm hydrogel layer. The dimensions were determined using SEM.[1] The modeling of the electric field intensity and distribution was performed using a conductivity of 2.2 mS/cm, which is the sum of the conductivity of a buffer containing 10 mM NaCl in 1x TBE (1.0 mS/cm) and that of 2% agarose hydrogel (1.2 mS/cm).[2,3] We used the Nernst-Planck equation to simulate the distribution of DNA and exchanges of ions on the chip with 1.5 VDC applied to the center electrode.[4,5] The effect of surface charge on the gel was assumed to be negligible due to the low density of DNA and high applied electric field.[6] The distributions of voltage, electric field and current were simulated by setting the central electrode to 1.5 V and other electrodes to ground. The Nernst-Plank equation was used to calculate the distributions of ions and DNA. The electric mobility of the Na^+ and Cl^- was set to $1.28x10^9$ m^2/s and $1.77x10^9$ m^2/s, respectively, according to the literature.[7] The net charge of a 17-base long DNA molecule was assumed to be -17*e*.

The results from the simulations are shown in Supplementary Fig. 5b-f. Supplementary Fig. 5b, c show the distribution of the electric field and field lines, and the potential profiles in both the vertical and horizontal directions around the center electrode. As can be observed, the area with the highest field strength is around the center electrode with an effective area (with > 0.6 V) of about ~100 μm, slightly greater than the physical size (80 μm) of the electrode. The distribution of histidine molecules

is shown in Supplementary Fig. 5d. Most histidine molecules are positioned near the center electrode with a profile slightly broader than those of simple inorganic ions, very likely due to its ability to change ionization state by the H^+ and OH^- ions produced by the electrochemical hydrolysis of water.[8] This ability to neutralize the H^+ and OH^- ions minimizes the electrochemical attack on other molecular species and the hydrogel layer. Supplementary Fig. 5e-f show the distributions of Na^+ and Cl^- ions.

Finally, the distribution of DNA molecules is shown in Supplementary Fig. 5g. A 17-base long ssDNA was used for the simulation and the effect of the gel matrix on the electrophoretic migration of the DNA was not considered. The ssDNA is concentrated at the positive electrodes after the application of the positive potential for only 90 s. In practice, the bit-encoding DNA molecules very likely hybridize to the encoding DNA template molecules already immobilized on the hydrogel surface and matrix upon encountering the gel layer during the migration toward the electrodes.

We also determined the Faradaic current distribution (Supplementary Fig. 6). The current mostly flows at the edges of the positive electrode. In our experiments, we found that the application of a high current could cause the detachment of the hydrogel layer from the surface due to electrochemical hydrolysis (Supplementary Fig. 7).

Optimization of electrophoretic transport and electric field-induced hybridization

Repetitive writing, reading, and logical operations are required to operate the DNA MLC-NVM systems. Ideally, random-access activation, writing by EFH and bitwise manipulations by EFD of the memory cells can be performed in parallel in a relatively small number of steps, and the operations are carried out using reactive DNA components in a solution over the entire memory array. In addition to operation speed, robust and reproducible operations of the system should also be taken into consideration. Guided by computational simulations, we experimented with key parameters, which include the applied electric potential, concentrations of DNA and buffers, to identity the working conditions. For the particular 100-site Nanogen microchips with individually addressable ring electrodes (80 µm in diameter, 200 µm spacing) covered under a 10 µm thick 2% agarose hydrogel layer, we found that efficient EFH could be performed in a buffer containing 50 mM histidine, 10 mM NaCl and 20 µM of DNA by applying 1.5 V to the electrodes for 1.5 minutes without electrochemical damage to the hydrogel layer. Some typical results are shown in Supplementary Fig. 8.

We also investigated the specificity of random access to the memory cells by addressing only certain cells in localized areas of the 100-site microchips. As described in the main text, a biotinylated dA_{25} DNA template was used to encode the data bit. A Cy3-labeled dT_{25} was used to write the data bit. The writing and reading operations were performed as described in the main text. Supplementary Fig. S5 shows the fluorescence images of three sub-arrays of memory cells on the same microchip. The sub-arrays were written and read one at a time.

Supplementary References

1. Krishnan R. et al. Interaction of nanoparticles at the DEP microelectrode interface under high conductance conditions, *Electrochem. Commun.* **11,** 1661–1666 (2009).
2. Kandadaia M.A., Raymond J. L. & Shawa G. J. Comparison of electrical conductivities of various brain phantom gels: developing a ‘brain gel model’. *Mater. Sci. Eng. C Mater. Biol. Appl.* **32,** 2664–2667 (2012).
3. Song Y., Sonnenberg A., Heaney Y. & Heller M. J. Device for dielectrophoretic separation and collection of nanoparticles and DNA under high conductance conditions. *Electrophoresis* **36,** 1107–1114 (2015).

4. Park H. M., Lee J. S. & Kim T. W. Comparison of the Nernst–Planck model and the Poisson–Boltzmann model for electroosmotic flows in microchannels. *J. Colloid and Interface Sci.* **315,** 731–739 (2007).
5. Dickinson E. J. F., Ekström H. & Fontes E. COMSOL Multiphysics®: Finite element software for electrochemical analysis. *Electrochem. Commun.* **40,** 71–74 (2014).
6. Maurer K. et al. Electrochemically generated acid and its containment to 100 micron reaction areas for the production of DNA microarrays. *PLoS ONE* **1,** e34 (2006).
7. Yang C. N. & Yang C. B. A DNA solution of SAT problem by a modified sticker model. *Biosystems* **81,** 1-9 (2005).
8. Martin E. J., Sadman K. & Shull K. R. Anodic electrodeposition of a cationic polyelectrolyte in the presence of multivalent anions. *Langmuir* **32,** 7747−7756 (2016).
